# Adhesion and aggregation properties of *Lactobacillaceae* strains as protection ways against enteropathogenic bacteria

**DOI:** 10.1007/s00203-022-02889-8

**Published:** 2022-04-27

**Authors:** Anna Zawistowska-Rojek, Anita Kośmider, Karolina Stępień, Stefan Tyski

**Affiliations:** 1 Department of Antibiotics and Microbiology, National Medicines Institute, Warsaw, Poland; 2 Department of Pharmaceutical Microbiology, Medical University of Warsaw, Warsaw, Poland; 3 Department of Biochemistry and Clinical Chemistry, Medical University of Warsaw, Warsaw, Poland; 4 Department of Cancer Biology, Maria Skłodowska-Curie National Research Institute of Oncology, Warsaw, Poland

**Keywords:** Adhesion, Aggregation, Caco-2, HT-29, *Lactobacillaceae*, Strains competence

## Abstract

The adhesion and aggregation are characteristic attributes of probiotic strains belonging to *Lactobacillaceae* genus. Due to these properties the host organisms can avoid colonisation of the intestinal tract by enteropathogenic bacteria. The presented research includes a comparison of the properties of various strains belonging to different *Lactobacillaceae* species and isolated from different sources The aim of this study was to investigate the ability of *Lactocaseibacillus rhamnosus, Lactiplantibacillus plantarum,* and *Lactobacillus* strains (*L. acidophilus, L. gasseri, L. ultunensis)* from probiotic products and clinical specimens to direct and competitive adherence to Caco-2 and HT-29 cell lines. Furthermore, the ability of lactobacilli and enteropathogenic bacteria, *E. coli, E. faecalis,* and *S.* Typhimurium, to auto- and co-aggregation was also investigated.

The results showed that all tested strains adhered to Caco-2 and HT-29 cell lines. Though, the factor of adhesion depended on the species and origin of the strain. *L. rhamnosus* strains showed a lowest degree of adherence as compared to *L. plantarum* and *Lactobacillus* sp. strains. On the other side both, *L. rhamnosus* and *L.* *acidophilus* strains reduced the pathogenic bacteria in competition adherence test most effectively. All tested lactobacilli strains were characterised by auto- and co-aggregation abilities, to various degrees. The properties of *Lactobacillaceae* strains analysed in this study, like adhesion abilities, competitive adherence, auto- and co-aggregation, may affect the prevention of colonisation and elimination of pathogenic bacteria in gastrointestinal tract.

## Introduction

According to the definition of the World Health Organization, probiotics are live microorganisms that, when administered in the appropriate amount, cause beneficial effects for the host organism (FAO/WHO 2002; Hill et al. 2014). Probiotic strains are applied in the production of various types of food products: fermented drinks, vegetables, and meats. The most common group of probiotics are lactic acid bacteria, especially strains from the family of *Lactobacillaceae* and *Bifidobacterium*, which belong to the gastrointestinal microbiota, and can be found most often in functional foods, medicinal products, dietary supplements, or medical devices (Monteagudo-Mera et al. 2019). The most important benefits of probiotic bacteria ingestion comprise stimulation of the immune system, production of antibacterial agents, regulation of the composition of the intestinal microbiota (Shehata et al. 2019), anti-mutagenic (eg. binding and transformation of mutagens or inhibition of the conversion of pro-mutagens to anti-mutagens) and anti-cancer properties (Prazdnova et al. 2022). Strains identified as a probiotic must demonstrate the ability to adhere to the mucous epithelial cells, cell lines, and should also be characterised by the ability to reduce the pathogenic microorganisms adhesion to the host cell surface (FAO/WHO 2002). The adhesion of microorganisms to the surface of intestinal cells is a way to extends the colonisation, which is important for the modulation of the immune response (Morita et al. 2002). Moreover, it may also influence the repair processes occurring in the injured intestinal mucosa (Morita et al. 2002). This behavior is one of the mechanisms that protect the host organism against pathogenic microorganism’s colonisation (Piątek et al. 2012).

Adhesion is a complicated process that enables microbes to adhere to other cells or surfaces (Duary et al. 2011; Paliwoda and Nowak 2017). The course of adhesion is influenced by many different factors, such as elements present in the cell wall, proteins, intestinal mucus, or environmental conditions (Paliwoda and Nowak 2017; Monteagudo-Mera et al. 2019). Initially, this process is based on physical interactions between surfaces, such as van der Waals forces or electrostatic interactions (Lewandowska et al. 2005; Behbahani et al. 2019). After attachment to the epithelial surface, the interaction between bacterial adhesins and receptors located in the epithelium plays an essential role (Lewandowska et al. 2005). The cell surface of the *Lactobacillaceae* bacteria contains capsular polysaccharides, teichoic and lipoteichoic acids, as well as various surface proteins (ex. mucin-binding protein, fibronectin-binding protein, collagen-binding protein) and lipoproteins. All of them allow the adhesion of these bacteria and the formation of biofilms on surfaces (Paliwoda and Nowak 2017; Archer et al. 2018; Monteagudo-Mera et al. 2019). On the other hand, structures and substances existing in the digestive tract, like mucin, extracellular matrix or lectin-like proteins, facilitate colonisation of probiotic strains (Grigoryan et al. 2018). The ability of *Lactobacillaceae* strains for auto-aggregation (an aggregation of bacteria belonging to the same strain) and co-aggregation (an aggregation of bacteria belonging to different species and strains) are related to the adhesion capacity (Kos et al. 2003; Collado et al. 2007; Hojjati et al. 2020). The natural colonisation process can be monitored and tested using cell lines. The colorectal adenocarcinoma cells, Caco-2 (non mucus secreting) and HT-29 (mucus secreting) are the most commonly used cell lines in the *Lactobacillaceace *in vitro adhesion studies (Sharma and Kanwar 2017). The Caco-2 cell line express morphological and functional differentiation in vitro and show characteristics of mature enterocytes. In turn, the HT-29 line shows a typical epithelial cell morphology, producing large amounts of mucus (Chauviere et al. 1992; Duary et al. 2011; Sharma and Kanwar 2017).

The resident gastrointestinal microbiota in vivo provides protection for the host against possible colonisation by the pathogenic bacteria (and play an important role in activating the immune system against these pathogens (Alp and Kuleasan 2019). Several reports have already documented the ability of probiotic lactobacilli and bifidobacteria to inhibit mucosa colonisation and invasion by pathogenic strains (Gopal et al. 2001; Ohashi and Ushida 2009). This may be associated with different mechanisms like: competition for nutrients and energy sources, which prevent pathogenic microorganisms’ growth and reproduction in the intestine (Cummings and Macfarlane 1997), production of antimicrobial substances by *Lactobacillaceae* strains (Chichlowski et al. 2007), competition for receptors of eukaryotic cells (Fonesca et al. 2021), immunomodulation (Fonesca et al. 2021), the intestinal barrier or co-aggregation abilities (Kos et al. 2003; Collado et al. 2007; Hojjati et al. 2020; Fonesca et al. 2021). If opportunities for pathogenic bacteria to adhere to host cells are reduced by the probiotic occupation of these sites, the incidence of infections may be reduced. According to Chapman et al. (2014), it is suggested that due to fewer sites being available to the pathogen, a greater reduction of infection occurrences is likely. Probably an application of probiotics as a method of prevention, can be more beneficial than medical treatment of infections. Many authors use three different variants of the study (competition assay, inhibition assay and displacement assay) to assess competitive exclusion. Li et al. (2008) showed that out of those three assays, the competition one showed the largest suppression of mucus adhesion both for the pathogens and lactobacilli. The displacement and inhibition assays exposed that, with respect to the addition order of the bacteria, those that were added latter had the predominance over bacteria that were already present. Many researchers have previously demonstrated protective effects against the attachment of a variety of enteric pathogenic bacteria, including *E. coli, S.* Typhimurium or *E. faecalis,* as the consequence of acidification with lactic acid (Ogawa et al. 2001; Markowiak and Śliżewska 2018), secreted nonacid products (Markowiak and Śliżewska 2017; Kerry et al. 2018), and interference with attachment to receptors or spaces, all of which may occur both directly and indirectly (Hirano et al. 2003).

Aggregation is associated with the surface of the bacterial cells and secreted substances, such as exopolysaccharides. These factors may play a significant role in the strength and speed of interactions between cells (Rajab et al. 2020). The ability to high auto-aggregation may also affect the longer duration of these strains in the digestive tract (Rajab et al. 2020). Rajab et al. (2020) suggest that auto- and co-aggregation properties may also depend on the length of bacterial cells. Longer cells present bigger surface areas, therefore, their aggregation is greater than in bacteria with short cells or spherical shape (Rajab et al. 2020). Some researchers have also reported that adherence and auto-aggregation of *Lactobacillus* cells are closely associated (Tuo et al. 2013; Celebioglu and Svennson 2018). On the other side, some scientists described, that strains with low ability to aggregation and co-aggregation may be characterised by a high degree of adhesion, which is opposite to the generally prevailing opinion (Alp and Kuleasan 2020).

The aim of the presented study was to investigate the possibility of protection of host organism against enteropathogenic bacteria colonisation, created by *Lactobacillaceae* strains derived from different sources. The characteristic of clinical strains that may have potential probiotic properties were compared with strains derived from available probiotic products and well characterized probiotic strain *L. rhamnosus* GG. The above goal was achieved through direct and competitive adherence of lactobacilli and selected standard enteropathogenic strains to enterocytes-like cell lines, the Caco-2 and HT-29. Moreover, auto- and co-aggregation of the lactobacilli and enteropathogenic bacteria, as processes impeding adherence, were investigated.

## Materials and methods

### Bacterial strains

The *Lactobacillaceae* strains used in this study were isolated from the probiotic products (dietary supplements, food for special medical purposes), present on the market in Poland, and from clinical material (swabs taken from cervix or anus of a healthy women, were collected at the Departament of Pharmaceutical Microbiology, Medical University of Warsaw, Poland). The following strains: *Lacticaseibacillus rhamnosus, Lactiplantibacillus plantarum, L. acidophilus, L. gasseri* and *L. ultunensis* were tested (Table 1). Moreover, *L.* *rhamnosus* GG (ATCC 53103), the most popular strain used in probiotic products (Capurso 2019), was used as a reference strain. All strains were identified by API 50 CHL tests (bioMérieux, France) and MALDI-TOF MS (ALAB Laboratory, Warsaw, Poland). The strains of the *Lactobacillaceae* family were grown on the De Man Rogosa and Sharpe Agar (MRS-Agar, Merck Millipore, Germany) in an atmosphere with 5% CO_2_ at 37 °C for 48–72 h.

**Table 1:** Strains from *Lactobacillaceae* genus used in the experiments

Strain symbol	API 50 CHL	MALDI TOF MS	Source of origin
LrA	*L. rhamnosus*	*L. rhamnosus*	Clinical isolate
LrB	*L. rhamnosus*	*L. rhamnosus*	Clinical isolate
LrC	*L. rhamnosus*	*L. rhamnosus*	Probiotic product
LrD	*L. rhamnosus*	*L. rhamnosus*	Probiotic product
LpE	*L. plantarum*	*L. plantarum*	Clinical isolate
LpF	*L. plantarum*	*L. plantarum*	Clinical isolate
LpG	*L. plantarum*	*L. plantarum*	Probiotic product
LpH	*L. plantarum*	*L. plantarum*	Probiotic product
LaI	*L. acidophilus*	*L. gasseri*	Clinical isolate
LaJ	*L. acidophilus*	*L. acidophilus*	Clinical isolate
LaK	*L. acidophilus*	*L. acidophilus*	Probiotic product
LaL	*L. acidophilus*	*L. ultunensis*	Probiotic product
Control	*L. rhamnosus*	*L. rhamnosus*	*L. rhamnosus GG* ATCC 53103

As exemplary enteropathogenic strains *E. coli* ATCC 8739*, E. faecalis* ATCC 29212 and *S. *Typhimurium ATCC 14028 were used. All strains were cultivated on Tryptic Soy Agar (Difco, Thermo Fisher Scientific, USA) at 37 °C for 24 h.

### Cell lines

The Caco-2 and HT-29 human colon carcinoma cell lines were purchased from American Type Culture Collection. Caco-2 cells were grown in Dulbeccoʼs modified Eagleʼs minimal essential medium DMEM (Gibco, Thermo Fisher Scientific, USA), supplemented with 10% heat-inactivated fetal bovine serum (Gibco), 1% nonessential amino acids solution (Sigma–Aldrich, USA), penicillin (100 U/mL) (Sigma–Aldrich) and streptomycin (100 μg/mL) (Sigma–Aldrich). HT-29 cells were cultured in the DMEM medium supplemented with 10% heat-inactivated fetal bovine serum, penicillin (100 U/mL) and streptomycin (100 μg/mL). Incubation of both cell lines was carried out at 37 °C in a 95% (v/v) humidified atmosphere with 5% (v/v) CO_2_. Caco-2 and HT-29 cultures were incubated for 20 days, to promote differentiation, and the medium was replaced every 24–48 h.

### Adhesion assay

For the adhesion assay Caco-2 cells and HT-29 cells, 20 days old cultures, were used. The cultures were grown until 85–95% of the surface areas covered. The medium was completely removed 24 h before adhesion assay with fresh DMEM medium without antibiotics. Prior to the assay, the cells were washed twice with phosphate-buffered saline (pH 7.4). Adhesion of various lactic acid bacteria (LAB) strains to Caco-2 and HT-29 cell lines was carried out with the method described by Lebeer et al. (2012) and Piątek et al. (2012) with necessary modifications. Acquired Caco-2 and HT-29 cells were grown at 37 °C in a humidified atmosphere of 5% CO_2_ in 12-well culture plates, starting from a density 4–5 × 10^4^ cells/cm^2^. After 20 days and before adhesion assay, the cells were washed with PBS buffer (Gibco) and counted using a haematocytometer chamber for the three different cultures wells, and the results were statistically evaluated.

LAB strains were incubated on the De Man Rogosa and Sharpe Broth (MRS-Broth, Merck Millipore) in an atmosphere with 5% CO_2_ at 37 °C for 20 h. After the incubation, the microbial culture was centrifuged at 4500×*g* for 10 min and the precipitate was washed twice with 0.9% NaCl. Bacterial cells were suspended in 1 mL of DMEM without antibiotics and fetal bovine serum to the density 1 × 10^7^ to 1 × 10^8^ CFU per mL, at a multiplicity of infection (MOI) of 500:1. The bacteria were then incubated with Caco-2 or HT-29 cells for 90 min at 37 °C in a 5% CO_2_ atmosphere.

Non-adhering bacteria were removed from both the Caco-2 and HT-29 cells by rinsing three times with PBS buffer. To release attached bacterial cells, the Caco-2 and HT-29 cultures were treated with a solution of 1% Triton X-100 (Sigma–Aldrich). The lysis was performed on ice for 10 min. The lysates were centrifuged at 4500×*g* for 10 min and the precipitate was washed twice with PBS. Finally, the precipitate was suspended in 1 mL of 0.9% NaCl, inoculated, and the number of adhered bacteria quantified according to the serial dilution method.

Serial decimal dilutions ranging from (10^– 1^ to 10^– 5^ CFU per mL) were prepared and plated out on the solid MRS medium. After the incubation of plates with 5% CO_2_ at 37 °C for 72 h, the number of *Lactobacillaceae* colonies was counted. The dose of bacteria used for the adhesion process, the number of adhering bacteria for used inoculum, and the number of adhering bacteria per 100 Caco-2 and HT-29 cells were also calculated. Three independent experiments were conducted and the results were statistically evaluated.

### Competitive adhesion of *Lactobacilli* and pathogenic bacteria

To study the competitive adhesion of pathogenic bacteria and *Lactobacillaceae* strains to Caco-2 and HT-29 cell lines, the competition assay tests were performed (Candela et al. 2008). Cell cultures and *Lactobacillaceae* strains with a density of 10^7^–10^8^ CFU/mL were prepared for the adhesion tests. In the case of pathogenic strains—*E. coli, E. faecalis* and *S*. Typhimurium, after an overnight cultivation (incubation in Tryptic Soy Broth, for 20 h, at 37° C in aerobic conditions), the suspensions with a density of 10^7^–10^8^ CFU mL (MOI 100:1) was prepared. For the competition adhesion assay, 1 mL of lactobacilli and 1 mL of pathogenic bacteria suspensions were added simultaneously to the same well of the plate with cell lines and then incubated at 37 °C with 5% CO_2_ for 90 min. After an incubation, all the non-adhered bacteria were removed by the method described above. Afterwards, the serial decimal dilutions ranging from (10^– 1^ to 10^– 5^ CFU/mL) were prepared and plated out on Tryptic Soy Agar for pathogenic bacteria. After incubation at 37 °C for 24 h in aerobic conditions, the number of bacterial colonies were calculated. The dose of bacteria used for the adhesion process, the number of adhering bacteria for used inoculum, and the reduction of adhering bacteria after incubation with *Lactobacillaceae* were calculated. Three independent experiments were conducted and the results were statistically evaluated.

### Auto-aggregation and co-aggregation

Auto-aggregation and co-aggregation analysis were performed in accordance with Kos et al. (2003) and Tuo et al. (2013) with modifications. *Lactobacillaceae* strains were grown for 20 h in a MRS broth in atmosphere enriched 5% CO_2_ at 37 °C. The bacteria were centrifuged at 5000×*g* for 20 min, washed twice with PBS and then re-suspended in PBS. The level of absorbance (A_600_) has been adjusted to a value of 0.25 ± 0.05 to standardize the number of bacteria (10^7^–10^8^ CFU/mL). The suspension (4 mL) was vortexed (A_initial_) and then incubated for 2 h at 37 °C (A_2h_). Auto-aggregation was expressed with equation:

$$1 - \frac{{A_{{2{\text{h}}}} }}{{A_{{{\text{initial}}}} }} \times 100$$

Bacterial suspensions for co-aggregation analysis were prepared with the same method as described for the auto-aggregation test. Two mL of both *Lactobacillaceae* (A_prob_) and pathogenic strain (A_path_) suspensions were mixed and then incubated at 37 °C without agitation. After two hours, the absorbance (A_mix_) was measured. Percentage of co-aggregation bacteria was determined as:

$$\left( {\frac{{\left( {A_{{{\text{path}}}} + A_{{{\text{prob}}}} } \right)/2 - A_{{{\text{mix}}}} }}{{(A_{{{\text{path}}}} + A_{{{\text{prob}})}} /2}}} \right) \times 100$$

In the equation presented above, the factors A_path_ and A_prob_ represent the absorbance of separate strains before incubation and A_mix_ represents the absorbance of mixed bacterial suspensions after 2 h of incubation.

### Statistical analysis

All results were expressed as the mean and standard deviation of three independent experiments. The one-way analysis of variance (one-way ANOVA), followed by post-hoc Tukey`s test for multiple comparisons. Data that showed no normal distribution were analysed using the non-parametric Kruskal–Wallis test followed by a pairwise comparison. *P* values < 0.05 were considered significant. Pearson correlation analysis was performed for adhesion to both cell lines versus auto-aggregation, with statistical significance at *p* < 0.05. Statistical analysis was conducted using SPSS software (version 28.0.1.0, IBM, IL, USA).

## Results

### *Lactobacillaceae* adhesion

**Fig. 1:**
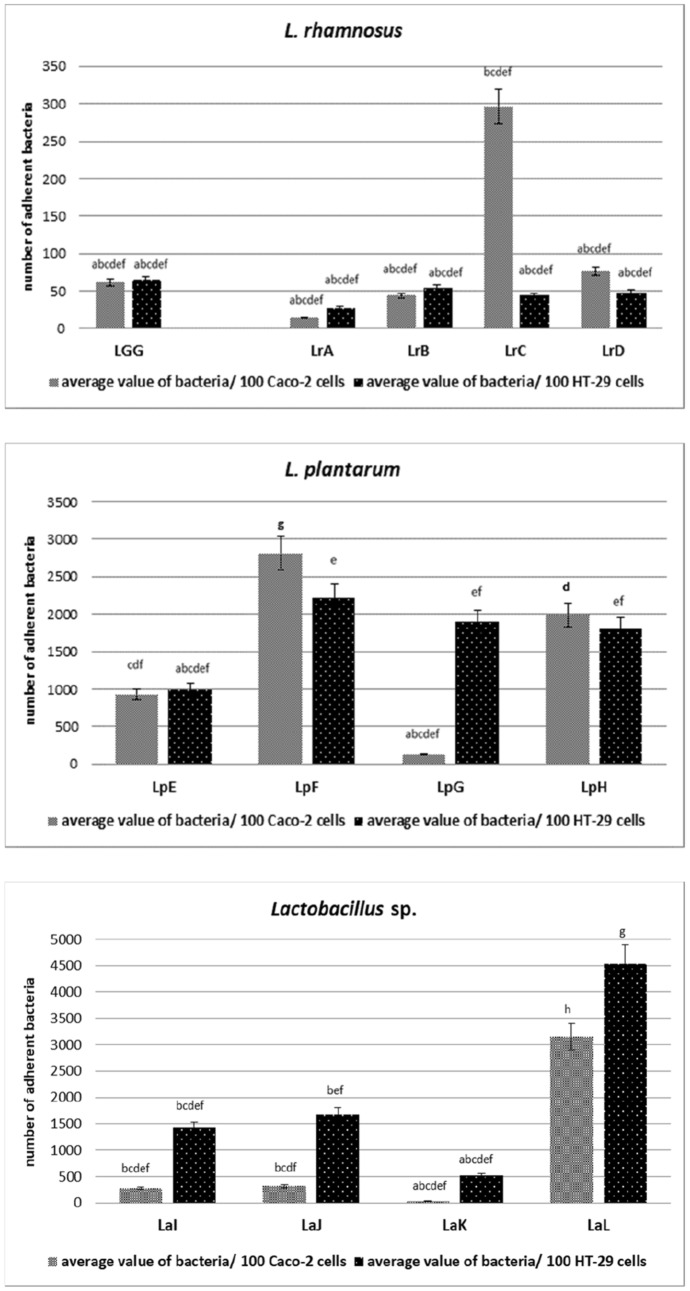
Adhesion of *Lactobacillaceae* strains to Caco- 2 and HT-29 cell lines, represents as average value and ± SD of adherent bacteria from 3 experiments per 100 Caco-2/HT-29 cells. Samples with different letters are significantly different (*p* < 0.05). Strain symbols are presented in Table 1

Adhesion of bacteria belonging to *Lactobacillaceae* family was performed on the Caco-2 and HT-29 tumor cell lines. The highest bacteria adhesion to the Caco-2 cell line (Fig. 1) was observed in the case of *L. plantarum* species (LpF—2800 CFU/100 cells Caco-2 and LpH—2000 CFU/100 cells Caco-2) and *L. ultunensis* (LaL—3150 CFU/100 cells Caco-2), the obtained values differed statistically (*p* < 0.05). In turn, the weakest adhesion was observed in the case of strains belonging to *L. rhamnosus* (LrA—15 CFU/100 cells Caco-2, LrB—45 CFU/100 cells Caco-2 and LrD—75 CFU/100 cells Caco-2), *L.* *plantarum* LpG—130 CFU/100 cells Caco-2 and *L.* *acidophilus* LaK—35 CFU/100 cells Caco-2. These strains did not show statistically significant differences compared to the control strain (LGG—62 CFU/100 cells Caco-2) (*p* < 0.05).

In the case of the second cell line, HT-29 (Fig. 1), the highest adhesion was observed with strains of *L. plantarum* (LpF—2200 CFU/100 cells HT-29, LpG—1850 CFU/100 cells HT-29 and LpH—1800 CFU/100 cells HT-29), *L. gasseri* (LaI—1420 CFU/100 cells HT-29), *L. acidophilus* (LaJ—1650 CFU/100 cells HT-29) and *L. ultunensis* (LaL—4500 CFU/100 cells HT-29), the obtained values differed statistically (*p* < 0.05), exception strains LpG and LpH (*p* > 0.05). The weakest adhesion was observed in the case of strains belonging to *L. rhamnosus* (LrA—30 CFU/100 cells HT-29, LrB—50 CFU/100 cells HT-29, LrC—40 CFU/100 cells HT-29 and LrD—50 CFU/100 cells HT-29). These strains did not show statistically significant differences compared to the control strain (LGG—64 CFU/100 cells HT-29) (*p* < 0.05).

**Fig. 2:**
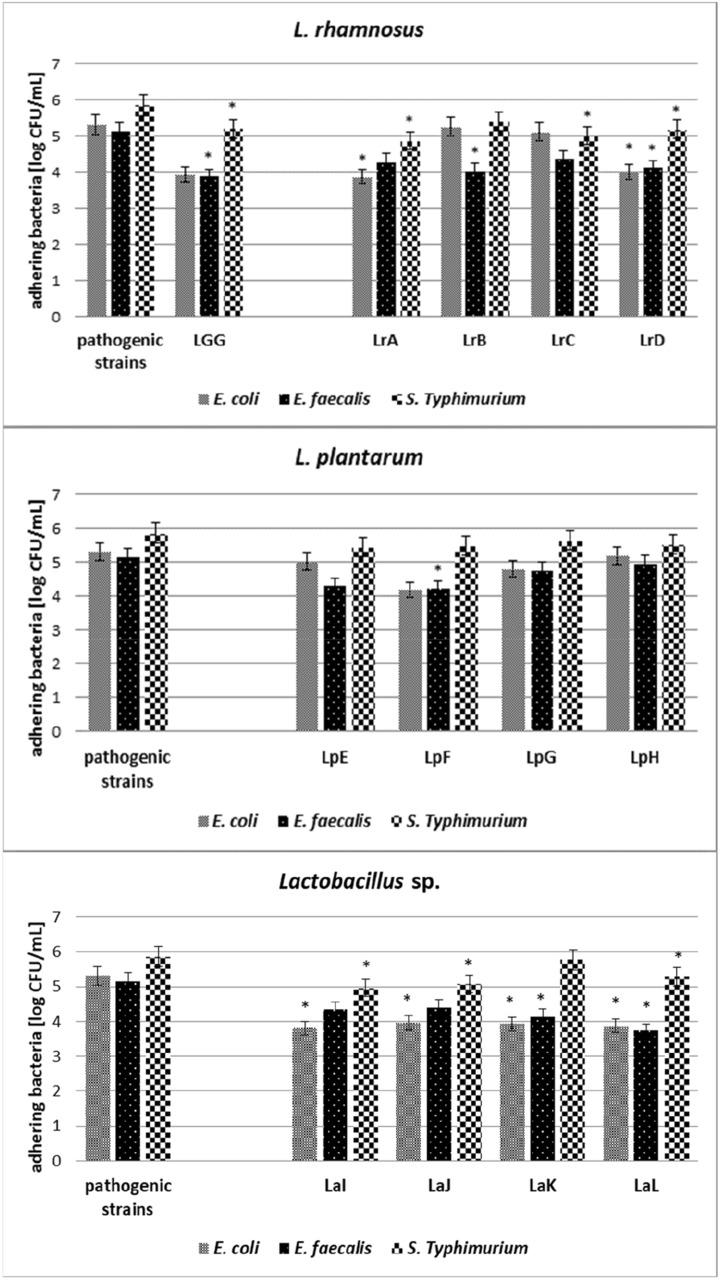
Competition adhesion test on Caco-2 cell line. Average value and ± SD of adherent bacteria from 3 experiments. *Significant differences among strains versus pathogenic bacteria. Strain symbols are presented in Table 1

**Fig. 3:**
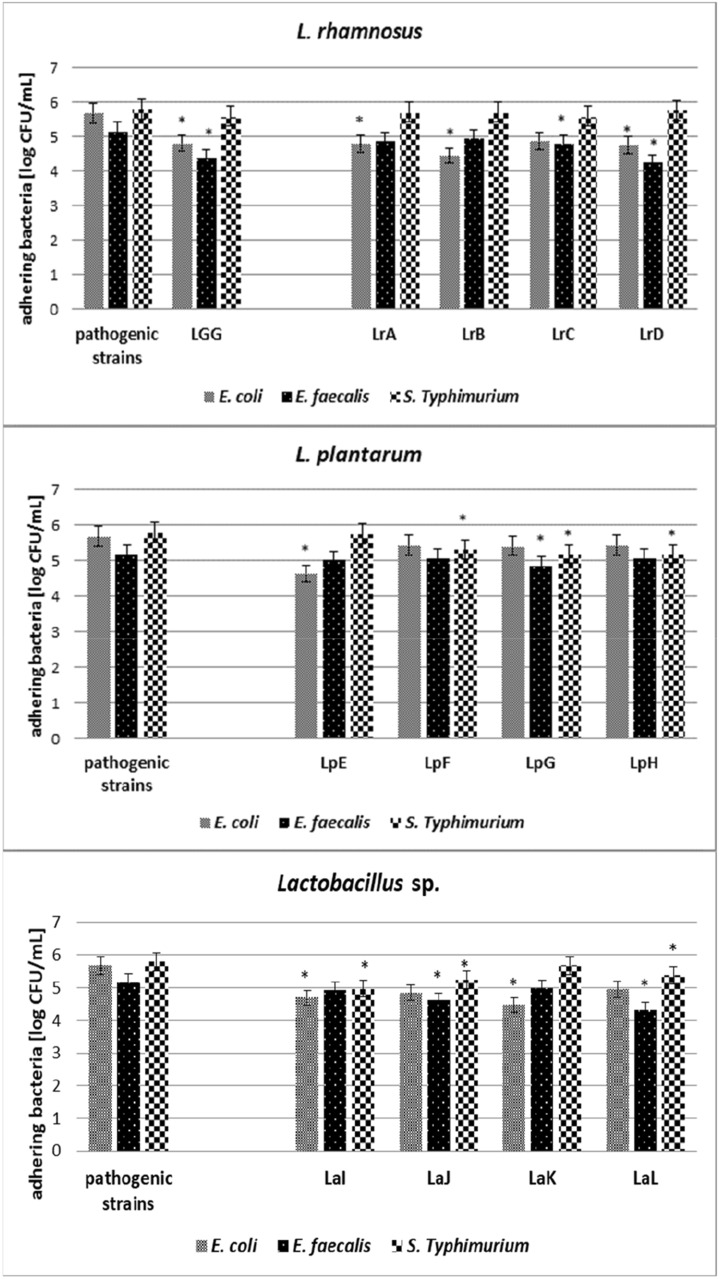
Competition adhesion test on HT-29 cell line. Average value and ± SD of adherent bacteria from 3 experiments. *Significant differences among strains versus pathogenic bacteria. Strain symbols are presented in Table 1

All tested *Lactobacillaceae* strains were characterised by the ability to reduce the adherence of pathogenic microorganisms—*S.* Typhimurium*, E. coli* and *E. faecalis* (Figs. 2 and 3). *Lactobacillus* sp. mostly inhibits the growth of *E. coli* strain (1.3–1.5 log CFU/mL), result was statistically significant, *p* < 0.05, on the Caco-2 cell line (Fig. 2). Moreover *Lacticaseibacillus* strains LrA and LrD also inhibit the adhesion of *E. coli* (1.4 log CFU/mL and 1.3 log CFU/mL, respectively, *p* < 0.05). On the other hand, statistically significant (*p* < 0.05) inhibition of the growth of *E. faecalis* was observed after incubation with the *Lacticaseibacillus* strains (LrB—1.1 log CFU/mL, LrD—1.0 log CFU/mL), *Lactiplantibacillus* LpF—0.9 log CFU/mL, and *Lactobacillus* (LaK—1.0 log CFU/mL and LaL—1.4 log CFU/mL). The statistically significant reduction (*p* < 0.05) of *S.* Typhimurium strains was manifested by *Lacticaseibacillus* (LrA—1.0 log CFU/mL, LrC—0.9 log CFU/mL, LrD—0.7 log CFU/mL) and *Lactobacillus* sp. strains (LaI—0.9 log CFU/mL, LaJ—0.8 log CFU/mL, LaL—0.6 log CFU/mL). The weakest reduction of *S*. Typhimurium adhesion was observed after incubation with *Lactiplantibacillus* strains (growth reduction 0.2–0.4 log CFU/mL, not statistically significant, *p* > 0.05).

In the studies focused on the HT-29 cell line (Fig. 3), the reduction of growth of *E. coli* about 1.0 log CFU/mL, statistically significant (*p* < 0.05), was observed after incubation with *Lacticaseibacillus* strains (LrA, LrB and LrD), *Lactiplantibacillus* LpE and *Lactobacillus* (LaI and LaK). The weakest reduction of adhesion was observed in the case of *Lactiplantibacillus* strains LpF, LpG, LpH—about 0.2–0.3 log CFU/mL (not statistically significant, *p* > 0.05). The reduction of *E. faecalis* was observed in case of *Lacticaseibacillus* (LrC—0.4 log CFU/mL and LrD—0.9 log CFU/mL), *Lactiplantibacillus* LpG—0.3 log CFU/mL and *Lactobacillus* (LaJ—0.5 log CFU/mL and LaL—0.8 log CFU/mL). In turn, statistically significant inhibition of the growth of *S*. Typhimurium strains was observed after incubation with strains of the genus *Lactiplantibacillus* (LpF—0.5 log CFU/mL, LpG—0.6 log CFU/mL, LpH—0.6 log CFU/mL) and *Lactobacillus* (LaI—0.8 log CFU/mL, LaJ—0.5 log CFU/mL, LaL—0.4 log CFU/mL), while *Lacticaseibacillus* strains did not inhibit the growth of *S*. Typhimurium at a statistically significant level.

### Auto-aggregation and co-aggregation

**Fig. 4:**
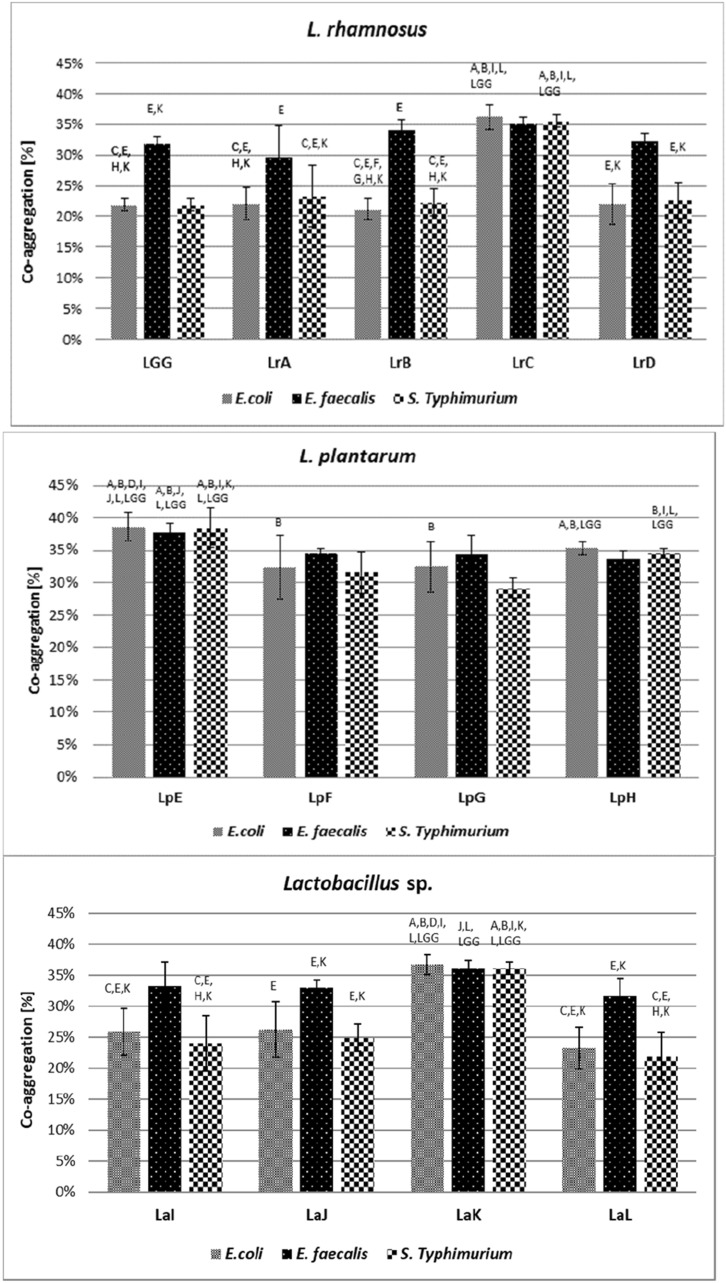
Co-aggregation of *Lactobacillaceae* strains with pathogenic bacteria after 2 h incubation at 37 °C. Average value and ± SD from 3 experiment. Different letters indicate the symbols of strains that differ statistically significantly (*p* < 0.05). Strain symbols are presented in Table 1

The level of auto-aggregation of *Lactobacillaceae* strains (Table 2) and co-aggregation between *Lactobacillaceae* and pathogenic bacteria (Fig. 4) after two hours of incubation were related to tested strains. The auto-aggregation values were in the range from 8.4% in clinical *L. plantarum* (LpE) isolate to 21.4% in *L. acidophilus* strain isolated from probiotic product (LaK). The auto-aggregation of *L. rhamnosus* GG strain was 13.1%, which is a value on a similar level as *Lacticaseibacillus* (LrB, LrC), *Lactiplantibacillus* (LpF, LpH) and *L. acidophilus* LaJ (*p* < 0.05). Pathogenic strains in the conducted study showed a lower ability to auto-aggregation, at the level from 5.5% for *E. faecalis* to 12.2% for *S.* Typhimurium.

**Table 2:** Auto-aggregation of *Lactobacillaceae* and pathogenic bacteria after 2 h incubation at 37 °C. Average value and ± SD from 3 experiments. Samples with different letters are significantly different (*p* < 0.05)

*Lactobacillaceae* genus	Strain symbol	Auto-aggregation ± SD
*Lactocaseibacillus rhamnosus*	LrA^ab^	10.1% ± 5.4%
	LrB^abcd^	14.6% ± 0.8%
	LrC^abcd^	15.0% ± 2.3%
	LrD^ab^	12.5% ± 2.8%
*Lactiplantibacillus plantarum*	LpE^a^	8.4% ± 1.5%
	LpF^abcd^	15.8% ± 2.9%
	LpG^cd^	20.5% ± 5.3%
	LpH^abcd^	15.4% ± 5.5%
*Lactobacillus* sp.	LaI^bcd^	18.0% ± 2.4%
	LaJ^abcd^	17.7% ± 1.4%
	LaK^d^	21.4% ± 1.7%
	LaL^abc^	12.3% ± 2.1%
Control strains	LGG^abcd^	13.1% ± 2.1%
	*E. coli*	11.8% ± 3.0%
	*E. faecalis*	5.5% ± 1.5%
	*S.* Typhimurium	12.2% ± 2.9%

The highest co-aggregation was observed between *Lactobacillaceae* and *E. faecalis* strains (approx. 33.8%), whereas the lowest was noticed in tests conducted with *S.* Typhimurium (approx. 28.7%) (Fig. 4). The clinical *L. plantarum* (LpE) was characterised by the lowest auto-aggregation (8.4%) and expressed the strongest co-aggregation ability with all tested probiotic strains, with values ranged 37.7–38.6% (depending on the pathogenic strains). The LGG strain was characterised by co-aggregation at the level of about 22% to *E. coli* and *S.* Typhimurium, and 32% to *E. faecalis*. The lowest co-aggregation was observed in *L. rhamnosus* (LrB) clinical strain in test with *E. coli*-only 21.2%. This strain was also characterised by low co-aggregation with *S. *Typhimurium (22.4%), weak adhesion to both Caco-2 and HT-29 cell lines and the lowest ability to reduce *E. coli* in competition test.

There was no correlation between auto-aggregation and adhesion to both tested cell lines of the tested strains (*p* > 0.05).

## Discussion

Probiotic strains, according to FAO/WHO guidelines, must present antagonistic properties against pathogenic microorganisms and present the ability to adhere to mucin and epithelial cells (FAO/WHO 2002). The pro-health effect of probiotic strains is manifested by hindering the colonisation of the gastrointestinal tract mucosa if it is caused by pathogenic strains. The most commonly used in adherence in vitro models, structurally and functionally similar to human enterocytes are colon adenocarcinoma cells (Duary et al. 2011). Good correlation between adhesion carried out in vitro*,* applying Caco-2 and HT-29 cell lines and adhesion in vivo, has been demonstrated in numerous studies conducted (Nowak and Motyl, 2017; Jose et al. 2017). Caco-2 cells have the ability to form a brush border, and create tight connections with each other (similarly to enterocytes). Those cell lines also have the ability to produce some enzymes (e.g., alkaline phosphatase, sucrase and aminopeptidase) and systems that transport substances from the lumen of the gastrointestinal tract directly into the bloodstream. As a result, cell lines used in this study show a functional similarity to the epithelium of the small intestine, imitating the natural in vivo conditions of the gastrointestinal tract (Hilgendorf et al. 2000). To be designated as probiotic, bacteria must adhere to mucosal epithelial cells lining the gut, which also depends on the number of bacteria added.

The value of adhesion *L. rhamnosus* GG strain obtained in this study (62 CFU/100 cells Caco-2 and 64 CFU/100 cells HT-29) was consistent with the results achieved by Gopal et al. (145 CFU/100 Caco-2 cell lines and 105 CFU/100 HT-29 cell lines) (Gopal et al. 2001). Deepika et al. (2009) conducted comparative studies of the adhesion of *L. rhamnosus* GG to Caco-2 cells under different growth conditions of cells. In the study, 20–200 bacteria per Caco-2 cell for 4–13 h cultures were used. Researchers suggested that differences in adhesion results may be due to different physiological conditions of bacterial cells, differences in bacterial cell culture and media conditions, and cell harvesting at different time points (Deepika et al. 2009). Moreover, the type of buffer and intensity of washing of non-adherent cells may also play a significant role (Hojjati et al. 2020). In our study *L. plantarum* strains were characterised by a much higher degree of adhesion than *L. rhamnosus.* The adhesion index of *L. plantarum* to Caco-2 cells depended on the strains (130–2820 CFU/100 cells Caco-2). The received results were on a similar level with the results achieved by Candela et al. (2008), amounting to 2530 CFU/100 Caco-2 cells for *L. plantarum* Bar10. The adhesion of *Lactobacillus* sp. strains, obtained in our study varied around the range of 517–4531 CFU/100 HT-29 cells, depending on the strains and their origin. Such variances in observed results was also seen in Lankaputhra and Shah (1998) research, where adherence of different *L. acidophilus* strains varied between 4 and 380 CFU/100 HT-29 cells.

In agreement with a previous study (Li et al. 2008), our results exposed that the adhesion ability of *Lactobacillaceae* strains to cell line was strains-specific and varied even within the same species, moreover, it could be associated with the strains origin, which is in line with the observations of other researchers (Sharma and Kanwar 2017; Rajab et al. 2020). These observations were aligned to the results seen in previous studies (Li et al. 2008; Mandal et al. 2016). Our observations also indicated the difference in adhesion index between two tested lines—Caco-2 and HT-29. The level of mucus secreted by the cells may play a significant role in adhesion (Sharma and Kanwar 2017).

The particular LAB strains in the present study were evaluated with regard to their ability to inhibit the adhesion of *E.* *coli,* *S.* Typhimurium and *E.* *faecalis*, to Caco-2 and HT-29 cells. The strongest inhibition of *E.* *coli* adherence was observed in the case of incubation with *L.* *acidophilus* strains on Caco-2 and HT-29 cells. Fonesca et al. (2021) reported inhibition of adhesion of *E.* *coli* in competition test by five different LAB strains on Caco-2 cell line at the significant level about 0.7 to 1.7 log CFU/mL. These authors reported also, that only two strains were able to reduce significantly *E. coli* strain on HT-29 cell line. Similar differences between the reduction of the number of pathogenic bacteria and the tested cell line were also noticeable in our work. In our study most of tested strains also reduce the adhesion of *E.* *coli* on Caco-2 cell line, at level about (0.5–1.5 log CFU/mL), only four strains probably were not able to reduce pathogenic strains (LrB, LrC, LpE, and LpH). In the case of HT-29 cell line only three strains of *L.* *plantarum* (LpF, LpG, and LpH) were characterised by very low reduction of *E.* *coli* cells adhesion.

Gopal et al. (2001) showed a 28–54% decrease of *E. coli* attachment to different epithelial cells with *L.* *acidophilus;* in the case of *L.* *rhamnosus*, the observed adherence reduction was in the range of 18–23%. All strains tested in our study demonstrated reduction of adhesion of pathogenic bacteria. The results suggest that the strains used in the presented study could prevent colonisation of the gastrointestinal tract by relevant pathogens such as *E. coli, S.* Typhimurium and *E.* *faecalis.*

The ability of *Lactobacillaceae* to aggregation can form a barrier and may exclude pathogenic strains from adhesion to gastrointestinal tract (Klopper et al. 2018). This property depends significantly on the incubation time (Piwat et al. 2015). Piwat et al. (2015) reported auto-aggregation of *L. rhamnosus* strains from the human oral cavity in the range more than 50% and *L. plantarum* about 50% after 24 h of incubation. Sophatha et al. (2015) also reported auto-aggregation of *L. rhamnosus* strains after 24 h incubation at the level of 55–60%. The results achieved by Grigoryan et al. (2018) were in the compartment of about 15% for *L. delbrueckii* subsp. *bulgaricus* to more than 70% to *L. helveticus* after 24 h incubation. The auto-aggregation of twenty LAB strains reported by Tuo et al. (2013) after 5 h incubation ranging from 24 to 41%, where the highest value has been reached by control *L. rhamnosus* GG strain. Collado et al. (2007) carried out auto-aggregation test focusing on to examine 2, 16, 20 and 24 h time periods. Results achieved in this test for *L. plantarum* was comparable to the results obtained in our study. The auto-aggregation of *L. plantarum* ATCC 14917 after 2 h incubation was reaching value about 25% (Wang et al. 2018) while for *L. plantarum* DM 69, after the same time of incubation, this value was 59% (Mohanty et al. 2019). In our study, for different *L. plantarum* strains this value ranged about 8.4–20.5%. D`Alessandro et al. (2021) tested auto-aggregation of vaginal lactobacilli strains. After 5 h of incubation some *L.* *gasseri* and *L. crispatus* strains achived the value of auto-aggregation above 90%, compared to the LGG control strain achieving auto-aggregation of 23% at the same time (D`Alessandro et al. 2021), while in our study, after 2 h incubation, this value for LGG strain reached about 13%. Kos et al. (2003) showed how the auto-aggregation of *L. acidophilus* strains were changing during 5 h of incubation, starting from about 25% after 1 h, to almost 70% after 5 h period. The same researchers tested also co-aggregation of *L. acidophilus* strains with pathogens—*E. faecium, E. coli* and *S. *Typhimurium. After 5 h of incubation the received results were as follow: 19%, 15% and 16%, respectively (Kos et al. 2003). Sophatha et al. (2020) tested co-aggregation of *Lactobacillus* strains after 24 h of incubation with pathogens such as enterotoxigenic *E. coli*, non-enterotoxigenic *E. coli*, *S. enterica*, ranges between 35 and 66%. Co-aggregation of different *Lactobacillaceae* strains with *E. coli* O157:H7 after 5 h incubation ranges between 21 and 32% (Sophatha et al. 2020). The co-aggregation results depend on the auto-aggregation properties between *Lactobacillus* and pathogen strains (Sophatha et al. 2020) and from the time of incubation (Piwat et al. 2015). In this study co-aggregation between *L. rhamnosus* and pathogenic strains, *E. coli* and *S*. Typhimurium, probable depends on the origin of the tested strain. In the case of clinical strains, the co-aggregation value ranged between 21–22% for *E. coli* and 22–23% for *S.* Typhimurium, in contrast to the strains isolated from probiotic products where these values were reached respectively 25–36% and 26–36%. Kowalska et al. (2020) reported that the co-aggregation rates may also depend on the source of pathogenic strains. The authors observed the highest co-aggregation for *L. rhamnosus* LOCK 1131 with *S.* Typhimurium ATCC 13311—84%, and the lowest for *L*. *casei* LOCK 1132 and *S*. Typhimurium ATCC 14028—11% (Kowalska et al. 2020).

## Conclusions

In conclusion, it seems that most of the *Lactobacillaceae* sp. strains may play a role in the protection of the gastrointestinal mucosa against colonisation with pathogenic strains, such as *E. coli, S.* Typhimurium or *E. faecalis*. Studies on the adherence of *Lactobacillaceae* strains and their competitive adherence to cell lines indicate the protective function of these strains. Similarly, the phenomenon of bacterial auto-aggregation and co-aggregation may reduce the degree of mucosa colonisation by pathogenic strains.

## Data Availability

All data generated and analysed during the study are included in the manuscript.
